# Co-Hydrothermal Carbonization of Polystyrene and Brewery Waste: Evaluation of Fuel Properties and the Effect of Blending Ratio and Temperature on Hydrochar Properties

**DOI:** 10.3390/polym18161940

**Published:** 2026-08-07

**Authors:** Monthakan Chanthip, Plaifah Sunprasit, Napat Kaewtrakulchai, Sutee Chutipaijit, Nuttapong Ruttanadech, Phongthorn Julphunthong, Tawat Suriwong, Pramote Puengjinda, Gasidit Panomsuwan, Achanai Buasri, Sappasith Klomklao, Masayoshi Fuji, Apiluck Eiad-Ua

**Affiliations:** 1 Department of Nanoscience and Nanotechnology, School of Integrated Innovative Technology (SIITec), King Mongkut’s Institute of Technology Ladkrabang, Bangkok 10520, Thailand; 2 Kasetsart Agricultural and Agro-Industrial Product Improvement Institute, Kasetsart University, Bangkok 10900, Thailand; 3 Department of Engineering, King Mongkut’s Institute of Technology Ladkrabang, Prince of Chumphon Campus, Chumphon 86160, Thailand; 4 Department of Civil Engineering, Faculty of Engineering, Naresuan University, Phitsanulok 65000, Thailand; 5 School of Renewable Energy and Smart Grid Technology, Naresuan University, Phitsanulok 65000, Thailand; 6 Deutsche Gesellschaft für Internationale Zusammenarbeit (GIZ) GmbH, Bangkok 10110, Thailand; 7 Department of Materials Engineering, Faculty of Engineering, Kasetsart University, Chatuchak, Bangkok 10900, Thailand; gasidit.p@ku.ac.th; 8 Department of Materials Science and Engineering, Faculty of Engineering and Industrial Technology, Silpakorn University, Nakhon Pathom 73000, Thailand; achanai130@gmail.com; 9 Department of Food Science and Technology, Faculty of Agro and Bio Industry, Thaksin University, Phatthalung Campus, Phatthalung 93210, Thailand; 10 Advanced Ceramic Center, Nagoya Institute of Technology, Tajimi 466-8555, Japan; fuji@nitech.ac.jp

**Keywords:** co-hydrothermal carbonization, synergistic effect: hydrochar properties, polystyrene, brewery waste

## Abstract

This study investigates the co-hydrothermal carbonization (Co-HTC) of polystyrene (PS) and brewery waste (BW) to produce high-quality hydrochar with enhanced fuel properties. Experiments were carried out with different PS-to-BW ratios and reaction temperatures to assess their impact on hydrochar yield, physicochemical properties, and combustion performance. The results showed that higher PS content notably enhanced both the hydrochar yield and higher heating value (HHV), demonstrating increased energy recovery potential. FTIR analysis revealed a gradual reduction in peak intensities with increasing temperature, indicating enhanced decomposition and structural transformation of functional groups. Examination of combustion properties demonstrated improved ignition and firing behavior, evidenced by reduced combustion durations and elevated combustion indices. Van Krevelen analysis showed that the co-hydrochars migrated toward the coal compositional region, supporting their potential as a sustainable alternative to conventional solid fuels. These findings underscore Co-HTC as a promising waste-to-energy strategy for the concurrent valorization of plastic and biomass waste streams.

## 1. Introduction

The rapid increase in municipal solid waste (MSW), particularly plastic waste, has become a major environmental challenge driven by urbanization and rising consumer demand [1]. Global plastic production has increased continuously since the 1940s, reaching a cumulative production of approximately 9.5 billion tons by 2020, while annual production exceeded 390 million tons in 2021 [2]. As plastic production continues to rise, the amount of plastic waste has also increased substantially, with global plastic waste generation doubling to approximately 353 million tons between 2000 and 2019 [2,3]. Among the various plastic wastes, polyethylene (PE), polyethylene terephthalate (PET), polyvinyl chloride (PVC), polypropylene (PP), and particularly polystyrene (PS) are widely used in short-life consumer products and packaging, resulting in large quantities of post-consumer waste. Despite ongoing recycling efforts, only a small fraction of plastic waste is effectively recycled, whereas the majority is landfilled, incinerated, or improperly discarded, causing long-term environmental pollution and the loss of valuable carbon resources [2,4]. In addition, conventional plastic production remains highly dependent on fossil resources and requires considerable energy input, highlighting the need for sustainable waste-to-resource technologies that can simultaneously reduce environmental impacts and recover valuable materials and energy [2,3].

Among commodity plastics, polystyrene (PS) is widely used in food packaging, disposable cups, and protective cushioning materials because of its low cost, light weight, and good mechanical properties [5]. However, its widespread use and poor degradability have resulted in the continuous accumulation of PS waste, creating serious environmental concerns. Owing to its carbon-rich aromatic structure, PS has attracted increasing attention as a promising feedstock for producing value-added carbon materials and for thermochemical conversion processes [6]. Recent studies have demonstrated the feasibility of co-hydrothermal carbonization (Co-HTC) of PS with lignocellulosic biomass. Sultana et al. [7] demonstrated the Co-HTC of styrofoam and sawdust for solid-fuel production, while Kaewtrakulchai et al. [8] investigated the Co-HTC of PS waste and maize stover followed by KOH activation for nanoporous carbon production. These studies demonstrate that PS can serve as an effective carbonaceous co-feedstock for hydrothermal conversion. However, investigations using brewery waste as the biomass feedstock have received limited attention, particularly regarding the combined effects of feedstock ratio and reaction temperature on hydrochar fuel properties.

Brewery waste (BW), also known as brewer’s spent grain, is one of the major by-products generated during beer production. Although BW has been utilized as animal feed [9] and incorporated into food products such as sausages [10] and bread [11], these applications consume only a portion of the total waste generated, and disposal remains a challenge for large-scale breweries. Because BW contains a high moisture content (70–78%), thermochemical conversion processes that require feedstock drying are generally less suitable. Instead, hydrothermal carbonization (HTC), which utilizes water as the reaction medium, has emerged as an attractive alternative for converting wet biomass into value-added solid fuel [12,13,14,15,16].

Hydrothermal carbonization (HTC) is typically conducted at 180–260 °C under autogenous pressure, where hydrolysis, dehydration, decarboxylation, condensation, and aromatization convert wet biomass into carbon-rich hydrochar [16,17]. Compared with pyrolysis and gasification, HTC is particularly suitable for high-moisture feedstocks because it eliminates the need for feedstock drying, utilizes water as the reaction medium, and operates under relatively mild temperatures, thereby reducing the overall energy demand for wet biomass conversion [18]. Moreover, HTC generally produces fewer gaseous emissions and volatile organic compounds (VOCs), as some nitrogen- and sulfur-containing species remain dissolved in the process water rather than being directly released into the atmosphere [19,20]. These advantages make HTC a promising thermochemical technology for converting wet biomass into value-added carbon materials and solid fuels [21,22]. Unlike conventional HTC, which generally processes a single feedstock, co-hydrothermal carbonization (Co-HTC) simultaneously converts two or more feedstocks, providing opportunities to improve hydrochar quality through interactions between different materials [17,21].

Recent studies have shown that Co-HTC can enhance the fuel properties of biomass–plastic mixtures through synergistic interactions during hydrothermal conversion [23,24]. For example, bamboo–PVC [25], cotton textile waste–PVC [23], pine sawdust–PVC [26], and corncob–polyurethane [27] systems have been reported to produce hydrochars with improved energy density, thermal stability, fuel ratio, and combustion performance compared with single-feedstock HTC. These improvements have been attributed to interactions between biomass-derived oxygenated compounds and plastic-derived intermediates, which promote dehydration, aromatization, structural reorganization, and carbon enrichment during Co-HTC. These synergistic interactions facilitate carbon retention and improve fuel quality compared with hydrochars produced from individual feedstocks [23,24,25,26,27].

Although previous studies have demonstrated the feasibility of Co-HTC for PS-containing waste mixtures, including styrofoam–sawdust [7] and PS waste–maize stover systems [8], the Co-HTC of brewery waste (BW) with PS remains insufficiently investigated. Unlike the lignocellulosic feedstocks examined in previous studies, BW is a moisture-rich industrial by-product with a complex biochemical composition that may influence hydrochar formation and the interactions occurring during Co-HTC. Consequently, the combined effects of reaction temperature and PS blending ratio on hydrochar yield, fuel properties, physicochemical characteristics, thermal behavior, and the synergistic interactions between BW and PS during hydrothermal conversion remain unclear.

Therefore, this study aims to systematically investigate the Co-HTC of BW and waste PS at different blending ratios and hydrothermal temperatures to address these knowledge gaps. The specific objectives were to (1) produce co-hydrochar from BW and PS mixtures under controlled Co-HTC conditions; (2) determine the effects of PS proportion and reaction temperature on hydrochar yield and fuel properties; (3) characterize the physicochemical and thermal properties of the resulting co-hydrochars; and (4) assess their potential as solid fuels using integrated fuel-quality and combustion-related indicators.

## 2. Materials and Methods

### 2.1. Materials

The brewery waste (BW) used in this study was obtained from a commercial brewery in Thailand. The BW was dried at 105 °C until a constant weight was achieved and then crushed and sieved to obtain a uniform particle size for subsequent experiments. Waste polystyrene (PS), mainly consisting of expanded polystyrene (EPS) disposable packaging materials, was collected from local waste collection sites in Thailand. The collected PS was manually sorted to remove non-polystyrene materials and other visible impurities before being cut into small pieces of similar size for subsequent use.

### 2.2. Co-Hydrothermal Carbonization

Brewery waste (BW) and polystyrene (PS) were used as feedstocks for the co-hydrothermal carbonization (Co-HTC) process. The total dry feedstock mass was fixed at 10 g for each experimental run. PS was incorporated into BW at 10, 20, 30, 40, and 50 wt%, with the remaining proportion consisting of BW, to investigate the effect of PS content on hydrochar properties. Deionized water was then added to obtain a solid-to-water ratio of 1:4 (*w*/*w*), and the mixture was thoroughly homogenized before being transferred into a 100 mL stainless-steel reactor (SRIVISAN KESARA Co., Ltd., Bangkok, Thailand). The reactor was tightly sealed and heated in a temperature-controlled oven at 220, 240, and 260 °C for a fixed residence time of 8 h. To ensure a consistent experimental design, the residence time was kept constant for all experimental conditions, allowing the effects of reaction temperature and PS content on hydrochar yield and physicochemical properties to be evaluated independently without the influence of an additional process variable. After the reaction, the reactor was allowed to cool naturally to room temperature before opening. The hydrochar recovered without washing and dried in a hot-air oven at 105 °C for 24 h prior to subsequent characterization.

The hydrochar mass yield was calculated using Equation (1):

(1) $$Mass\;yield\;(\%)=\frac{Mass\;of\;dried\;hydrochar}{Mass\;of\;untreated\;dry\;}\times 100$$

where the mass of dried hydrochar and untreated dry feedstock are expressed on a dry weight basis.

The energy yield of the hydrochar was calculated using Equation (2):

(2) $$Energy\;yield\;(\%)=\;\frac{Hydrochar\;yield\times {HHV}_{hydrochar}}{{HHV}_{feedstock}}$$

### 2.3. Sample Characterization

The higher heating value (HHV) of the samples was determined using a bomb calorimeter (C5000, IKA-Werke GmbH & Co. KG, Staufen, Germany) in accordance with international standard testing methods, based on the amount of heat released during complete combustion. The surface chemical functional groups were characterized by Fourier Transform Infrared Spectroscopy (Spectrum Two FTIR with UATR accessory, PerkinElmer Inc., Waltham, MA, USA), with spectra recorded in the wavenumber range of 4000–400 cm^−1^ to evaluate changes in chemical structure following sample preparation. The elemental composition of carbon (C), hydrogen (H), and nitrogen (N) was quantified using a CHN/O elemental analyzer (FlashSmart, Thermo Fisher Scientific Inc., Waltham, MA, USA). The surface morphology and microstructural features of the samples were examined using Scanning Electron Microscopy (Quanta 250, FEI Company, Hillsboro, OR, USA) at various magnifications. The crystalline structure was investigated by X-ray diffractometer (XRD) (SmartLab, Rigaku Corporation, Akishima, Tokyo, Japan), with diffraction patterns collected over an appropriate 2θ range to identify crystalline phases and the degree of amorphous character. The thermal decomposition behavior and combustion characteristics were assessed by Thermogravimetric Analysis (TG 209 F3 Tarsus, NETZSCH-Gerätebau GmbH, Selb, Germany) under a controlled atmosphere, by continuously monitoring changes in sample mass as a function of temperature and time.

(3) Fix carbon=100−(Ash+Volatile matter)

(4) $$Fuel\;ratio=\frac{Fixed\;carbon}{Volatile\;matter}$$

## 3. Results and Discussions

### 3.1. Effects of Different Co-HTC on Co-Hydrochar

**Figure 1:**
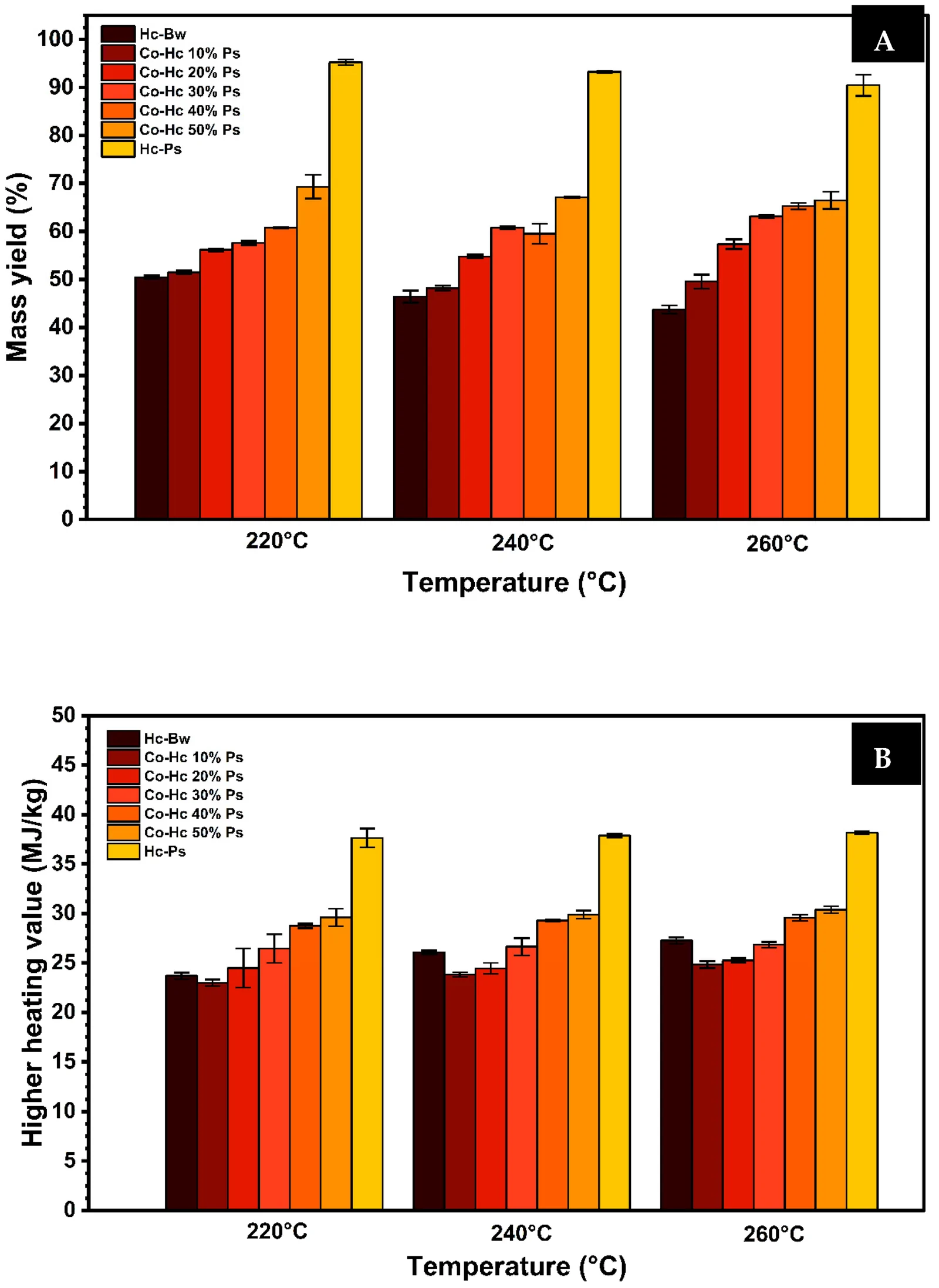

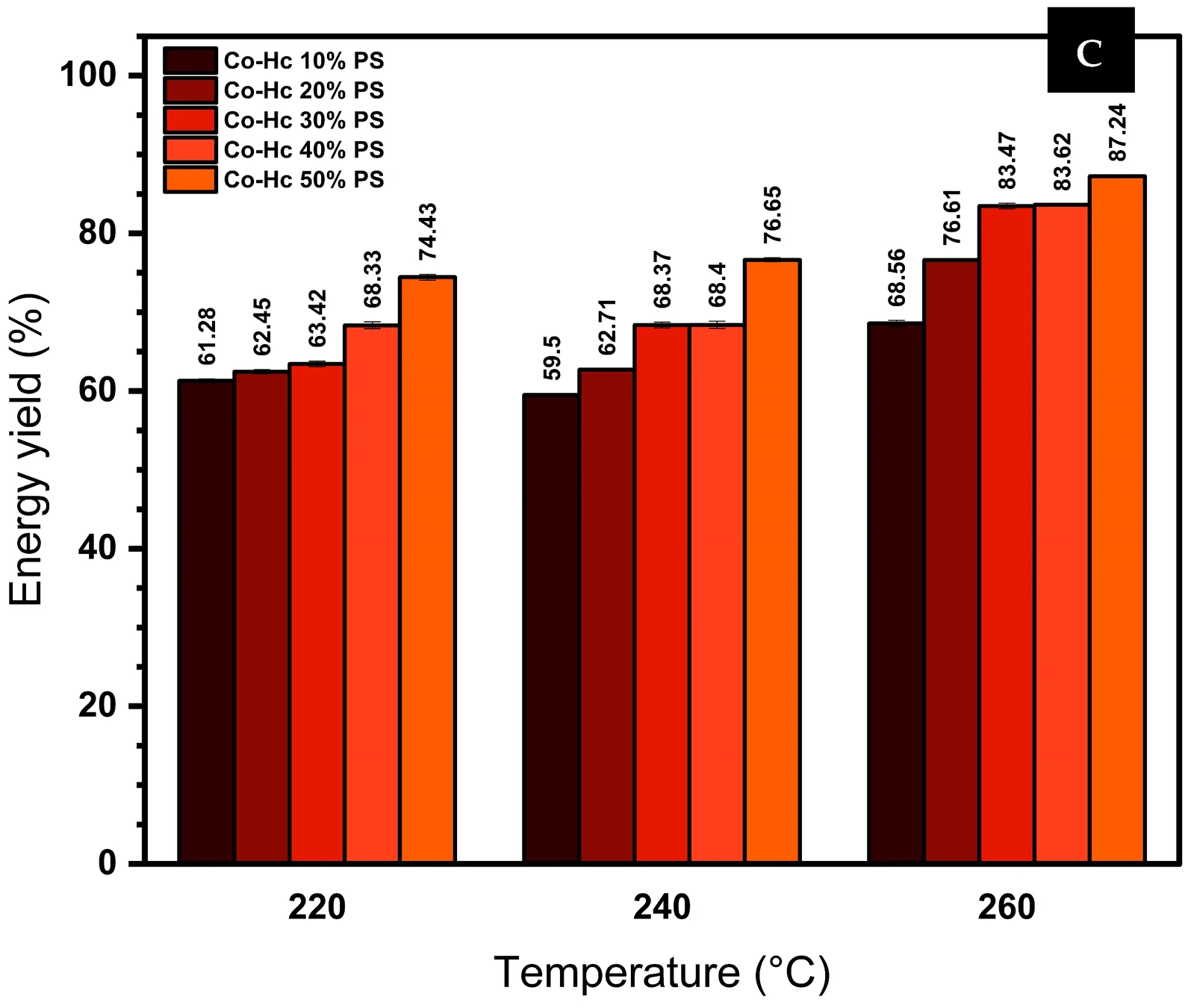
Effects of different Co-HTC on co-hydrochar (**A**) mass yield, (**B**) higher heating value (HHV), and (**C**) energy yield.

Figure 1A shows the co-hydrochar yield as a function of Co-HTC temperature. With increasing temperature, the overall hydrochar yield decreased. At a 50 wt% PS ratio and temperatures of 220, 240, and 260 °C, the hydrochar yields were 68.9%, 67.1%, and 66.2%, respectively, indicating a gradual reduction in solid product yield with increasing reaction severity. The decrease in hydrochar yield can be attributed to the progressive decomposition of biomass components during hydrothermal carbonization. Hemicellulose begins to decompose at approximately 180 °C, producing soluble polysaccharides that are subsequently hydrolyzed into monosaccharides. As the reaction temperature increased, these intermediates underwent dehydration, decarboxylation, and polymerization reactions, generating liquid- and gas-phase products that reduced the hydrochar yield [28,29]. Cellulose exhibited similar behavior but generally decomposed at higher temperatures (above 240 °C), promoting polymerization and the formation of aromatic structures that contributed to hydrochar formation through the development of aromatic clusters. Lignin, which is more thermally stable than hemicellulose and cellulose, decomposes over a broader temperature range through gradual depolymerization and condensation reactions. These reactions promote the formation of aromatic carbonaceous structures and contribute to carbon retention in the resulting hydrochar [29,30]. In contrast, PS primarily decomposes through depolymerization to styrene monomers, followed by the formation of intermediates such as benzyl alcohol, benzaldehyde, and benzoic acid [31]. These intermediates subsequently undergo dehydration and decarboxylation reactions, while cleavage of the C–C backbone generates free radicals that participate in secondary reactions [31,32]. Compared with lignocellulosic biomass, PS exhibits relatively higher thermal stability under HTC conditions because of its hydrocarbon-rich aromatic structure. Consequently, increasing the PS content resulted in higher hydrochar yields, indicating greater carbon retention in the solid phase during the Co-HTC process.

**Figure 2:**
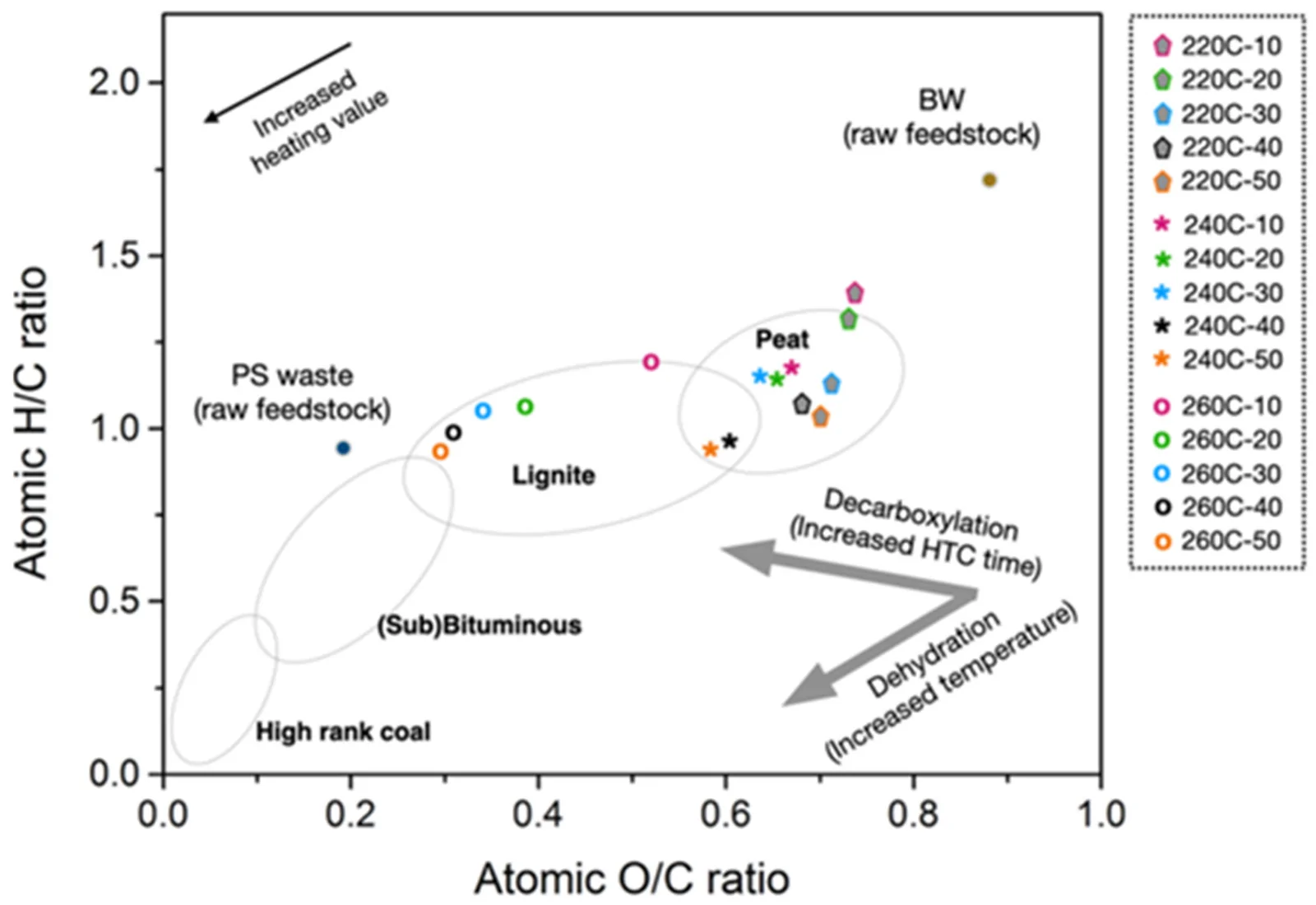
Van Krevelen diagram of hydrochar of brewery waste blended with polystyrene at different ratios via co-hydrothermal carbonization process at 220–260 °C.

The high heating value (HHV) is an important parameter for evaluating the fuel quality of hydrochar. As shown in Figure 1B, the HHV of the Co-HTC hydrochar was substantially higher than that of the raw materials, demonstrating that the Co-HTC process effectively improved the energy content of the solid product. At a 50 wt% PS ratio, the HHV increased by 37.49%, 37.81%, and 38.13% at 220, 240, and 260 °C, respectively. The differences in HHV among the investigated temperatures (220–260 °C) were relatively small, whereas increasing the PS content produced a slight but consistent increase in HHV. The increase in HHV can be attributed to the carbon-rich aromatic structure and high hydrocarbon content of PS, which promoted carbon enrichment during Co-HTC. Although partial depolymerization and C–C bond cleavage occurred at elevated temperatures, only a limited fraction of carbon was lost through decarboxylation and dehydration reactions [32,33], allowing more carbon to be retained in the hydrochar. This observation is consistent with the enhanced fuel characteristics of the hydrochar and suggests that incorporating PS into BW improved the carbonization behavior during the Co-HTC process. Furthermore, the increase in HHV is consistent with the decrease in the H/C and O/C atomic ratios (Figure 2), indicating progressive dehydration and decarboxylation and a higher degree of carbonization with increasing PS content and reaction temperature. To further evaluate the overall energy retention of the Co-HTC products, the energy yield was calculated by considering both the hydrochar yield and HHV (Figure 1C). As the PS content increased, the energy yield also increased at all investigated temperatures. At 220 °C, the energy yield increased from 61.28% for the hydrochar containing 10 wt% PS to 74.43% for that containing 50 wt% PS. A similar trend was observed at 240 °C, where the energy yield increased from 59.50% to 76.65%, while the highest values were obtained at 260 °C, increasing from 68.56% to 87.24% as the PS content increased from 10 to 50 wt%. Although increasing the reaction temperature resulted in a slight reduction in hydrochar yield, the simultaneous increase in HHV compensated for this decrease, resulting in improved overall energy retention in the solid product. Consequently, the hydrochar produced at 260 °C with 50 wt% PS exhibited the highest energy yield, indicating that this condition provided the most efficient retention of the feedstock energy in the solid phase. Similar relationships between hydrochar yield, HHV, and energy yield have been reported for hydrothermal carbonization of carbon-rich feedstocks [30,34].

### 3.2. Evaluation of Fuel Properties via Proximate Analysis

The proximate analysis results (dry basis) of the raw feedstock and co-hydrochar samples are presented in Table 1, including volatile matter (VM), fixed carbon (FC), ash content, and fuel ratio (FC/VM). The Co-HTC process markedly altered the fuel characteristics of the feedstock through the removal of volatile components and carbon enrichment of the solid product. A substantial reduction in VM was observed after Co-HTC, decreasing from 61.48 wt% in the raw BW to 9.81–13.31 wt% in the co-hydrochar samples. This reduction is mainly attributed to the decomposition of hemicellulose and cellulose together with dehydration and decarboxylation reactions, which released volatile compounds into the liquid and gaseous phases during hydrothermal carbonization [26]. Unlike the hydrochar produced solely from BW, the fixed carbon (FC) content of the co-hydrochar remained within the range of 10.66–15.85 wt%. Although the FC values did not increase substantially, the ultimate analysis (Table 1) showed a pronounced increase in carbon content accompanied by a marked decrease in oxygen content, indicating progressive carbonization and the formation of more condensed aromatic structures during the Co-HTC process [30]. These compositional changes explain the higher HHVs obtained for the co-hydrochar, demonstrating that the improvement in fuel quality was primarily associated with elemental composition rather than fixed carbon alone.

The fuel ratio (FC/VM) increased from 0.48 for the raw BW to 1.60 for the Co-HC260 sample. This increase resulted primarily from the significant reduction in volatile matter, which occurred more rapidly than changes in fixed carbon content. A higher fuel ratio generally indicates improved combustion characteristics and a greater degree of carbonization [34]. This interpretation is consistent with the higher HHVs and the lower H/C and O/C atomic ratios presented in Figure 2, which indicate progressive dehydration, decarboxylation, and carbon enrichment during the Co-HTC process.

The ash content increased from 8.93 wt% in the raw BW to 74.26–79.47 wt% in the co-hydrochar samples. This increase mainly reflects the relative concentration of inorganic constituents after the extensive removal of organic matter during hydrothermal carbonization and is expressed on a dry-weight basis rather than indicating the formation of additional mineral phases [15]. Although the increased ash content may reduce the combustible fraction of the fuel, the overall fuel characteristics of the co-hydrochar were enhanced, as evidenced by the higher HHV, increased fuel ratio, and lower H/C and O/C atomic ratios.

### 3.3. Quality Assessment of Raw Feedstock and Co-Hydrochar

Figure 2 shows the Van Krevelen diagram, in which the molar hydrogen-to-carbon (H/C) and oxygen-to-carbon (O/C) ratios are widely used to evaluate the degree of carbonization and coalification of carbonaceous materials [17,34]. A progressive decrease in both H/C and O/C ratios generally indicates the occurrence of dehydration and decarboxylation reactions together with the development of more aromatic carbon structures during hydrothermal carbonization [17,34]. The H/C and O/C ratios of lignite and bituminous coal are included as reference materials to evaluate the degree of the hydrochar products. For the co-hydrochars produced from brewery waste and polystyrene at different mixing ratios, a clear distinction was observed between the raw BW and the Co-HTC products. As confirmed by the elemental analysis (Table 1), increasing the reaction temperature from 220 to 260 °C progressively increased the carbon content while decreasing the oxygen content. This compositional evolution is consistent with the downward and leftward shift observed in the Van Krevelen diagram, reflecting enhanced dehydration and decreasing dehydration during the Co-HTC process. Consequently, the O/C ratio decreased markedly with increasing reaction temperature, while the H/C ratio also declined, indicating an increase in aromaticity and carbonization.

Furthermore, increasing the PS content contributed to a further reduction in the H/C and O/C ratios, indicating the formation of carbon-rich structures with fuel characteristics closer to those of low-rank coal. At 260 °C and 50 wt% PS, the co-hydrochar exhibited H/C and O/C ratios approaching those of lignite, indicating a higher degree of coalification than the raw BW.

### 3.4. Functional Group Assessment of the Co-Hydrochar and Raw Feedstocks

**Figure 3:**
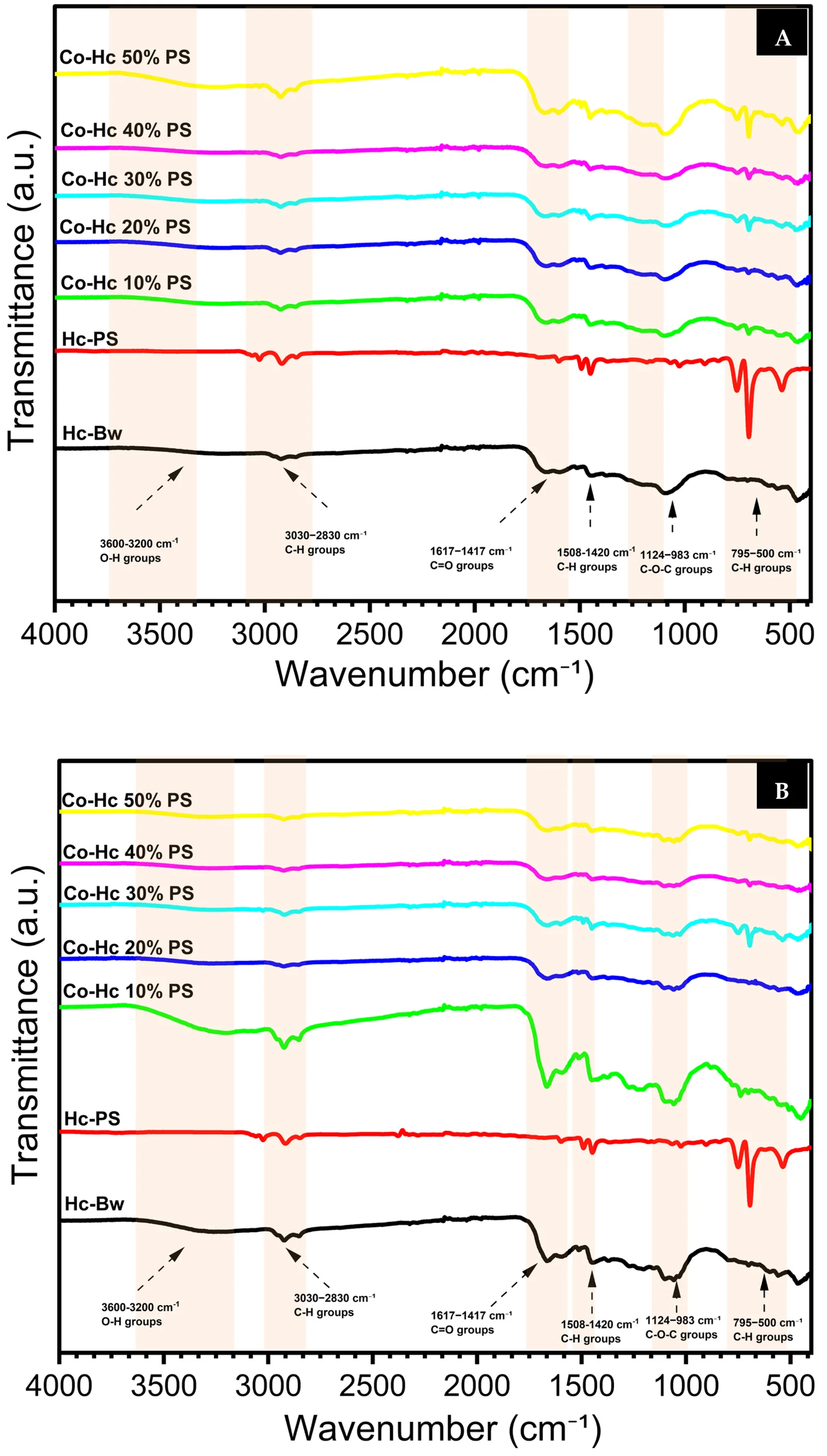

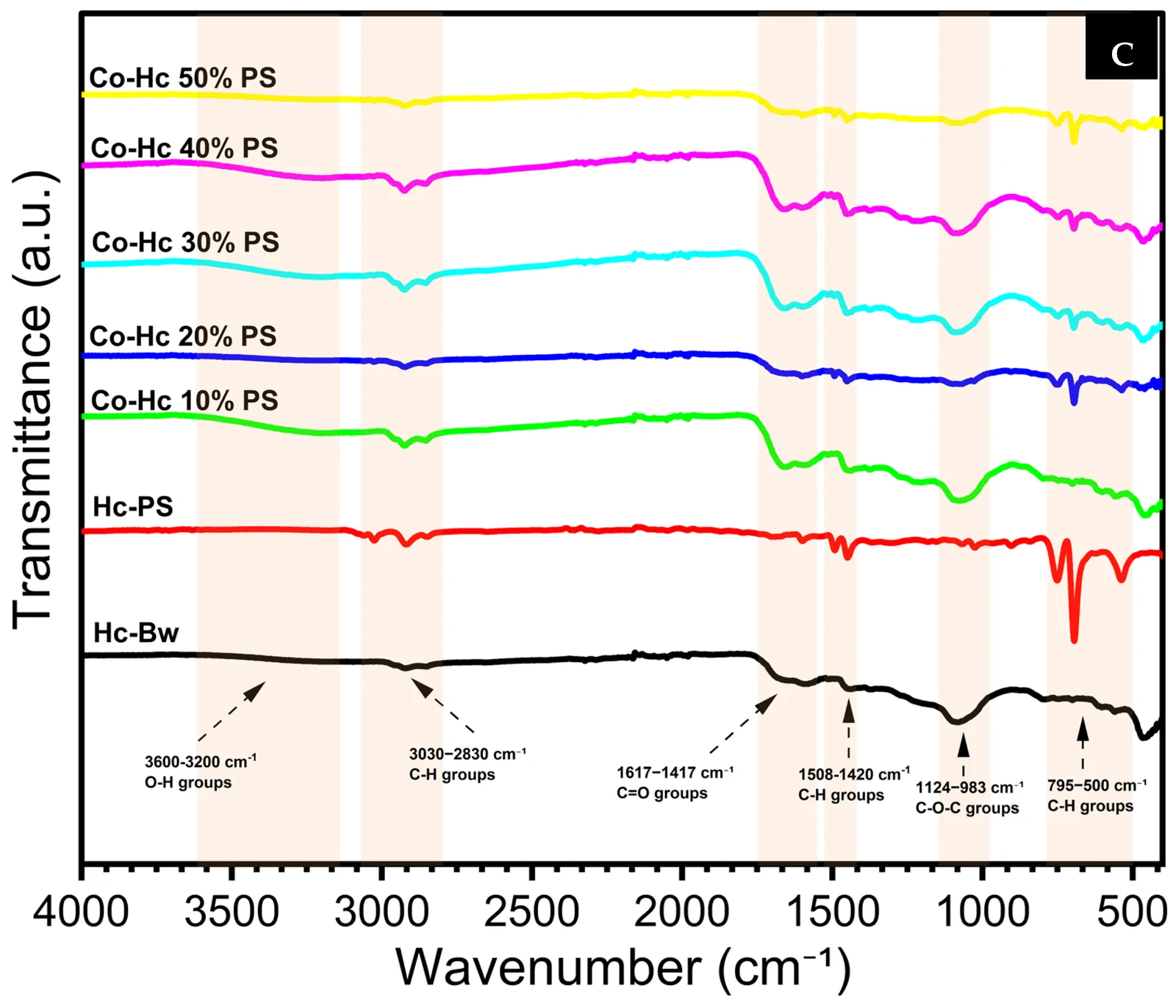
FT-IR spectra of brewery waste blended with polystyrene at different ratios via co-hydrothermal carbonization process (**A**) at 220 °C, (**B**) at 240 °C and (**C**) at 260 °C.

Figure 3 presents the FT-IR spectra of the raw materials (BW and PS) together with the co-hydrochars produced at different PS blending ratios under Co-HTC conditions. The spectra were recorded in the range of 4000–400 cm^−1^ using the transmittance mode. The co-hydrochars exhibited absorption bands characteristic of both BW and PS, indicating that structural features from both feedstocks were retained after the Co-HTC process.

The broad absorption band at 3600–3200 cm^−1^ is assigned to the stretching vibration of hydroxyl (–OH) groups associated with lignocellulosic components in BW. The absorption bands observed at 3030–2830 cm^−1^ correspond to aliphatic C–H stretching vibrations derived from cellulose and hemicellulose. The region between 1617 and 1417 cm^−1^ is attributed to aromatic C=C and carbonyl (C=O) stretching vibrations, representing lignin-derived aromatic structures as well as the aromatic ring structure of PS [20,31]. Additional absorption bands at 1508–1420 cm^−1^ were also observed, which are associated with aromatic C–H vibrations. Furthermore, the absorption region at 1124–983 cm^−1^ corresponds to C–O–C stretching vibrations of cellulose and hemicellulose, whereas the bands in the 795–500 cm^−1^ region are attributed to aromatic C–H bending vibrations originating from PS [31,32]. As shown in Figure 3, the intensity of the oxygen-containing functional groups, particularly the –OH and C–O–C bands, gradually decreased with increasing reaction temperature, indicating progressive dehydration and decarboxylation during the Co-HTC process. In contrast, the aromatic bands in the regions of 1617–1417 and 795–500 cm^−1^ became more distinguishable as the PS proportion increased, suggesting a greater contribution of aromatic carbon structures from PS to the resulting co-hydrochar. The reduction in the overall FT-IR peak intensity at higher temperatures indicates the decomposition of surface oxygen-containing functional groups and the formation of more condensed carbonaceous structures. This observation agrees well with the elemental analysis and the Van Krevelen diagram (Figure 2), which showed increased carbon content together with lower oxygen content and decreased H/C and O/C atomic ratios as the reaction temperature increased, confirming the progressive carbonization of the co-hydrochars [20].

### 3.5. Morphology of Hydrochar

**Figure 4:**
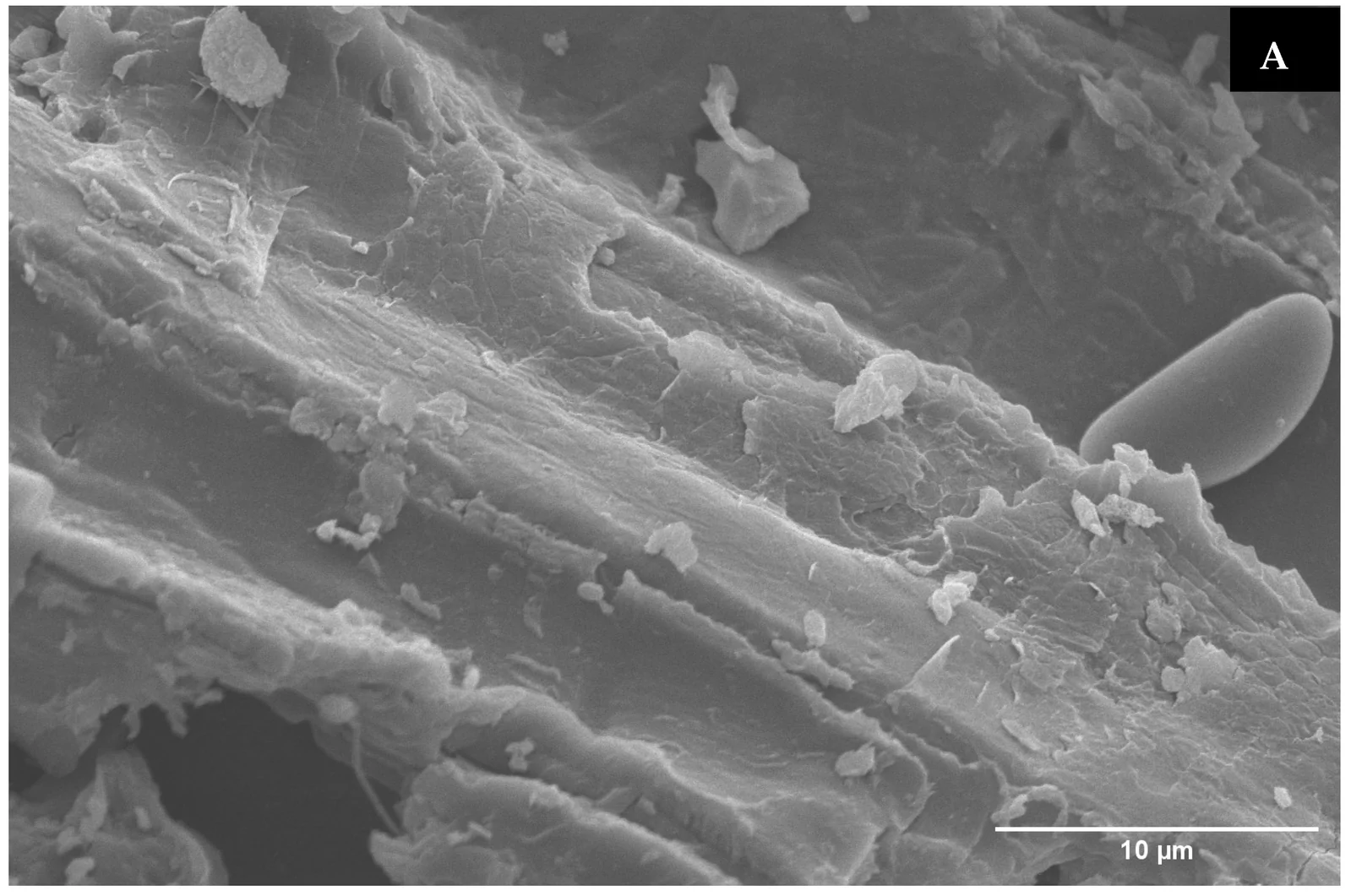

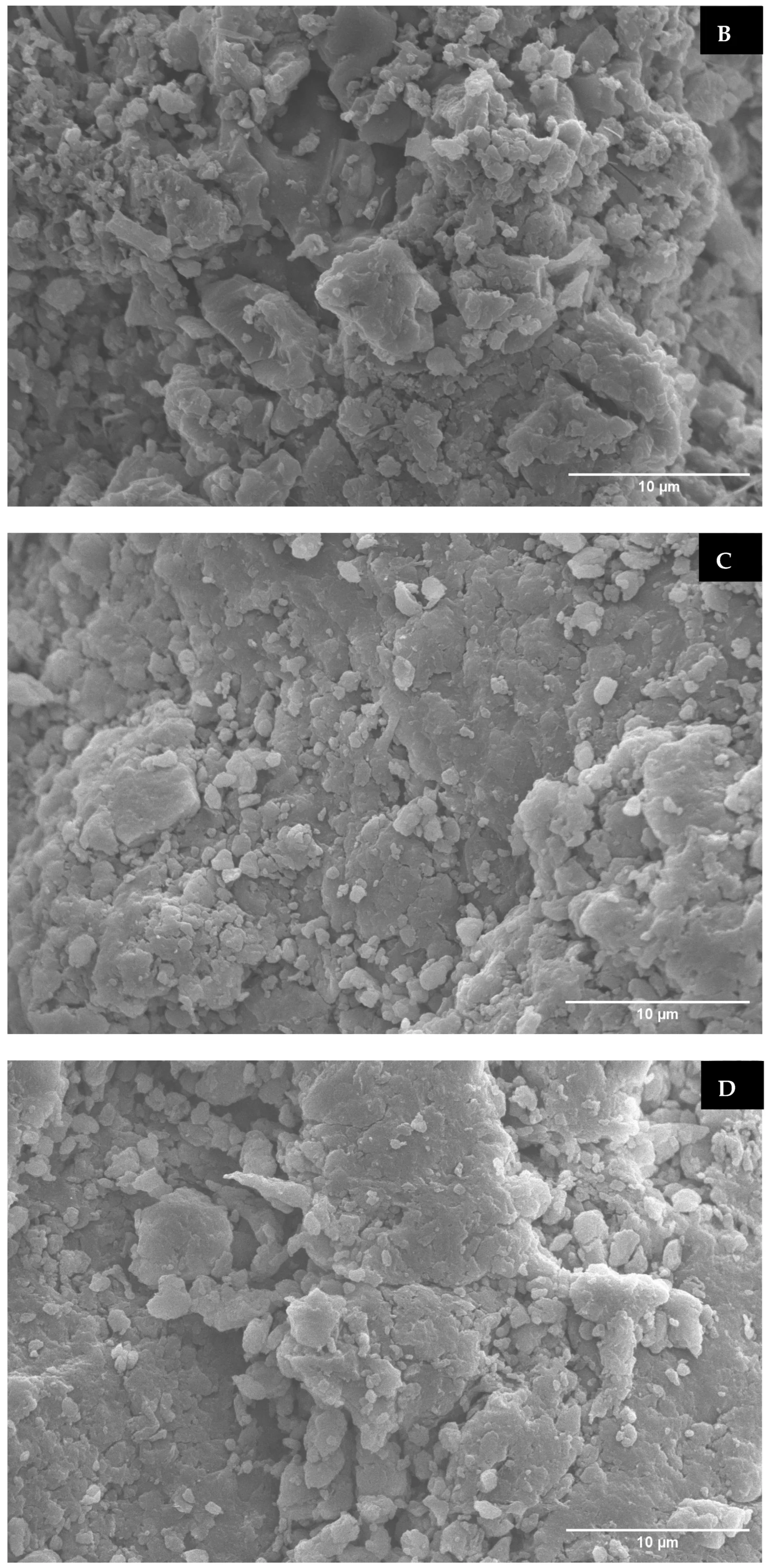

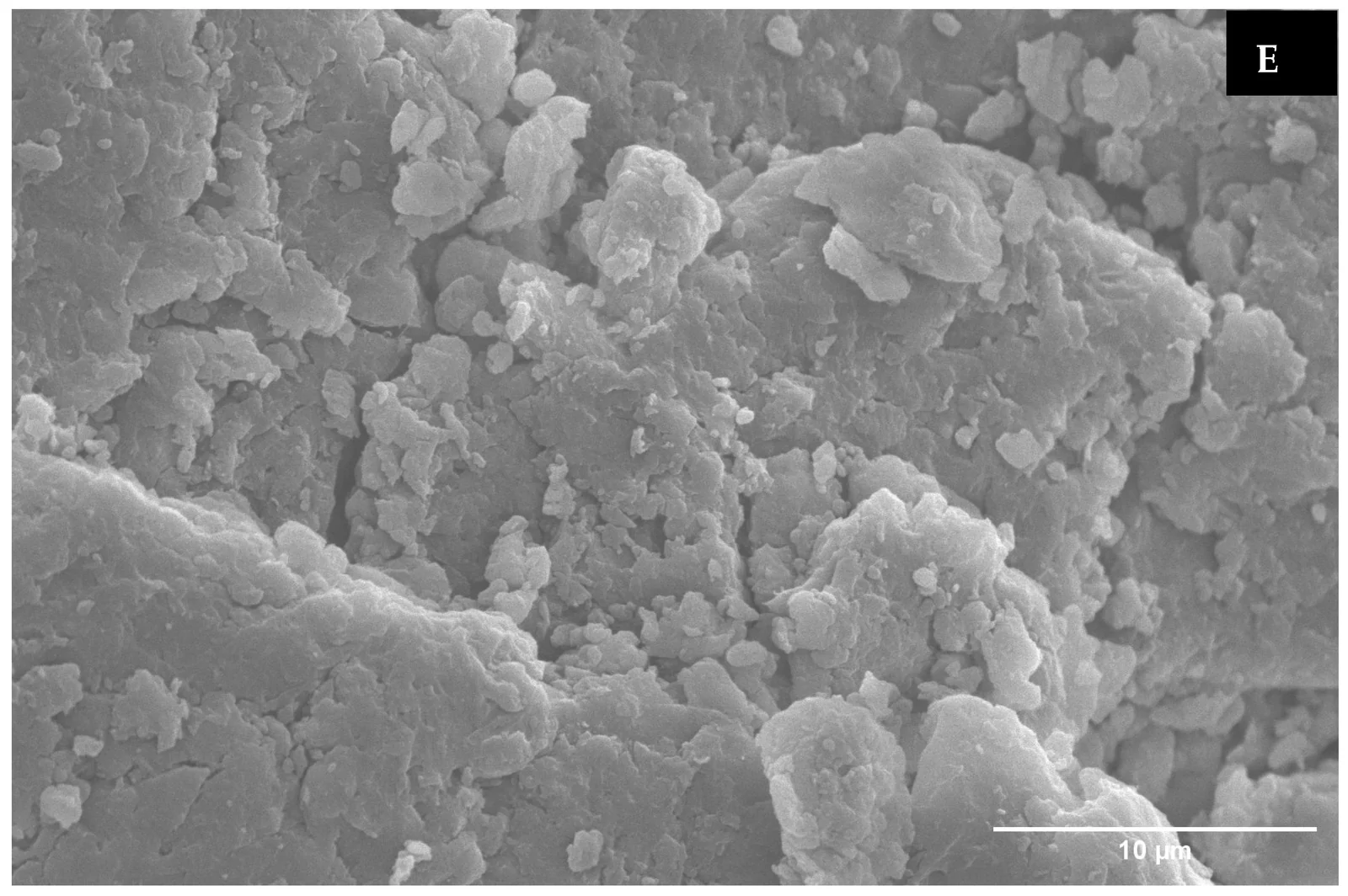
SEM micrographs of brewery waste blended with polystyrene at different ratios via co-hydrothermal carbonization process. (**A**) Raw BW, (**B**) at 200 °C, (**C**) at 220 °C, (**D**) at 240 °C and (**E**) at 260 °C.

The surface morphology of the co-hydrochars was examined by scanning electron microscopy (SEM), as shown in Figure 4, to evaluate the influence of reaction temperature on the structural evolution of the hydrochars during the Co-HTC process. The SEM images revealed distinct morphological changes with increasing reaction temperature, reflecting the progressive transformation of the BW–PS mixture into a more condensed carbonaceous structure. At 200–220 °C, the hydrochars retained a partially fibrous morphology with obvious fracture and disruption of the biomass fibers. These structural changes are attributed to the hydrolysis of ester and ether linkages within the lignocellulosic matrix, resulting in the decomposition of the plant cell wall and fragmentation of the fibrous structure [16]. The preservation of some fibrous features at these temperatures indicates that carbonization was still incomplete during the early stage of the Co-HTC process. As the reaction temperature increased to 240–260 °C, the hydrochars exhibited a more compact morphology accompanied by pronounced particle agglomeration. Under these conditions, PS softened and partially decomposed, enabling it to coat and bridge adjacent carbonized biomass particles. Consequently, a denser and more interconnected carbonaceous matrix was formed. This observation is consistent with previous studies reporting that plastic-derived phases can promote the formation of compact hydrochar structures during co-hydrothermal carbonization [35].

At higher magnification, numerous carbonaceous spherical particles were observed on the hydrochar surface, particularly in the samples produced at higher temperatures. These microspheres are commonly attributed to secondary char generated through the condensation and polymerization of water-soluble intermediates, such as 5-hydroxymethylfurfural (5-HMF) and furfural, formed during the hydrothermal decomposition of lignocellulosic biomass. The formation of these spherical particles is consistent with the LaMer nucleation model, which describes the nucleation and subsequent growth of particles under supersaturated conditions [30]. The progressive transition from fractured fibrous structures to dense agglomerated particles with increasing reaction temperature is consistent with the results of the elemental analysis, FT-IR spectra, and Van Krevelen diagram, all of which indicate enhanced dehydration, decarboxylation, aromatization, and carbonization during the Co-HTC process.

### 3.6. Crystalline and Amorphous Structures of Co-Hydrochar

**Figure 5:**
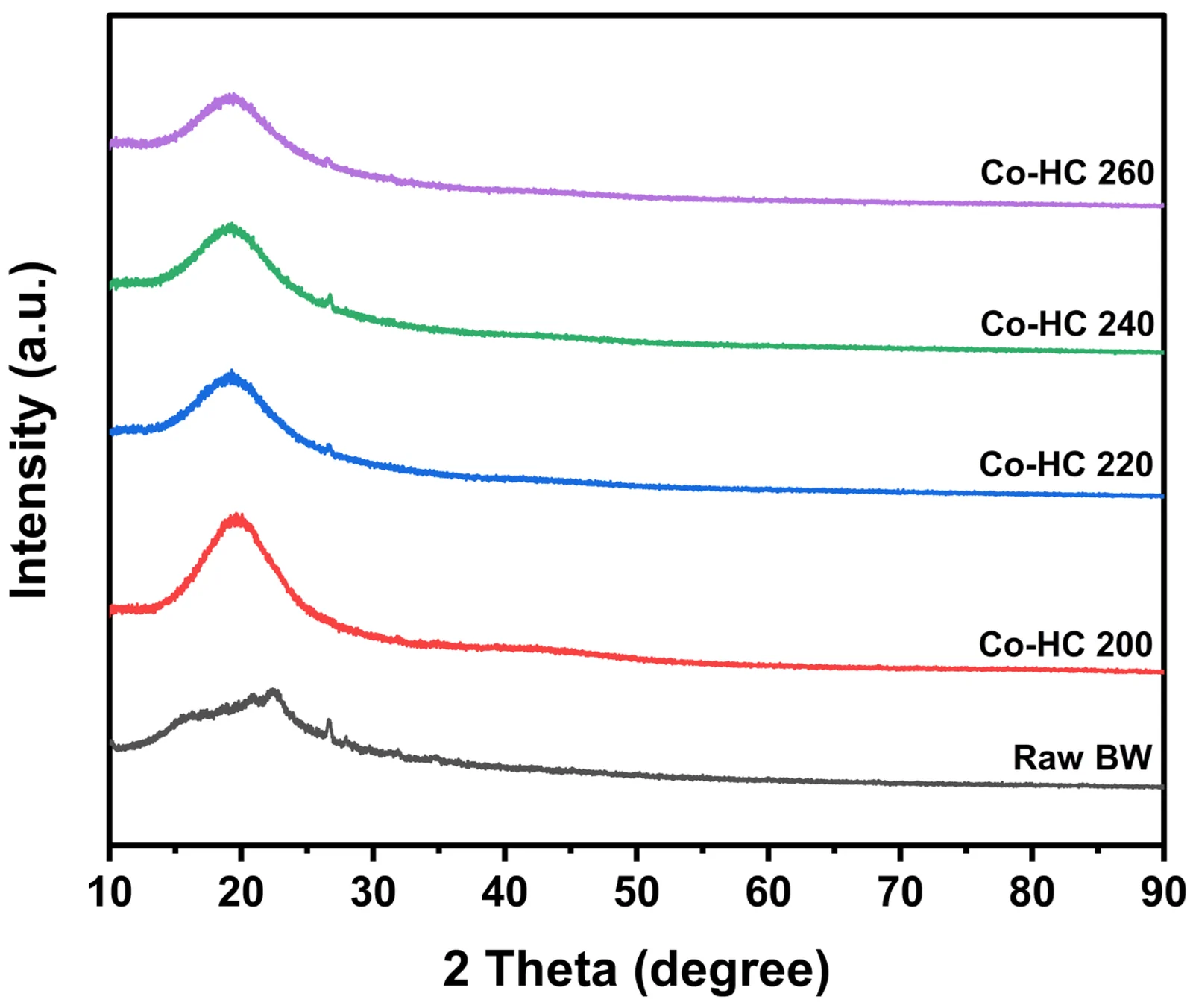
XRD patterns of brewery waste and co-hydrochar via co-hydrothermal carbonization process.

Figure 5 presents the X-ray diffraction (XRD) patterns of the co-hydrochar produced from BW and PS at a 1:1 ratio. A broad diffraction peak centered at approximately 2θ = 20° was observed, indicating that the co-hydrochar predominantly consisted of an amorphous carbonaceous structure with a low degree of structural ordering. This broad diffraction feature is commonly attributed to the overlapping of disordered aromatic carbon layers associated with the (002) plane of turbostratic carbon rather than to the presence of well-crystallized graphitic carbon [36]. The absence of sharp crystalline diffraction peaks suggests that the carbon framework formed during the Co-HTC process remained largely disordered. This structural characteristic is consistent with the hydrothermal carbonization of lignocellulosic biomass, where dehydration, decarboxylation, and condensation reactions promote the formation of amorphous aromatic networks instead of highly graphitized carbon structures [20,34]. Similar broad diffraction peaks in the 20–30° region have been reported for hydrochars and biochars derived from various biomass feedstocks, including rice husk, and are generally assigned to the (002) reflection of disordered aromatic carbon layers [36].

Compared with previously reported co-pyrolysis systems, the incorporation of PS did not result in the formation of additional crystalline carbon phases, indicating that the carbonaceous framework remained predominantly amorphous under the present Co-HTC conditions. A previous study on the co-pyrolysis of corn stover and PS also reported that the addition of PS influenced the development of carbon microstructures, although the resulting carbon remained largely disordered [37]. Therefore, the XRD results obtained in this study suggest that the co-hydrochar produced from BW and PS possesses a predominantly amorphous carbon structure, which agrees well with the FT-IR results and the elemental analysis indicating progressive carbonization during the Co-HTC process.

### 3.7. Combustion Properties of Co-Hydrocha

**Figure 6:**
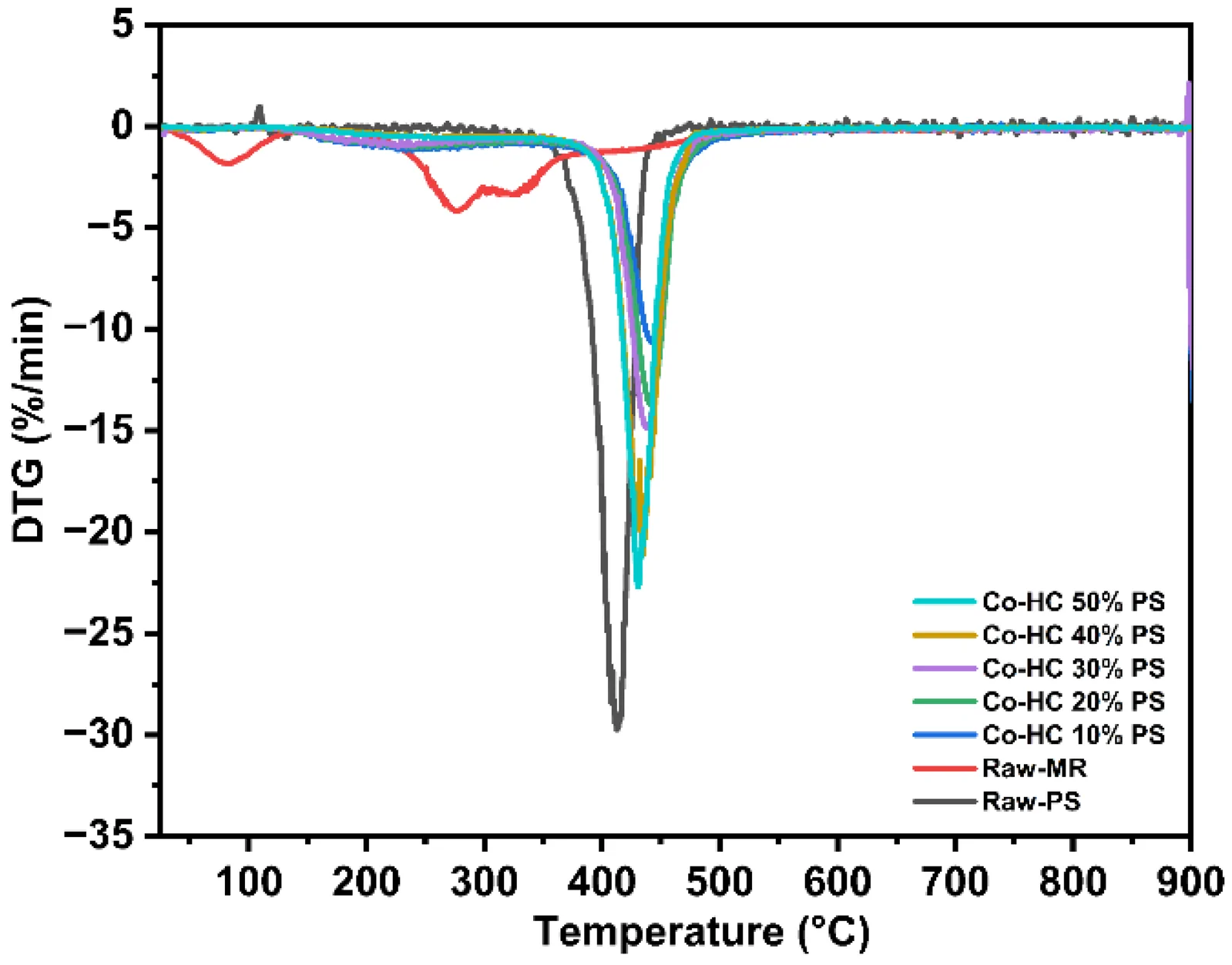
DTG (%/min) profile of the raw feedstocks and hydrochars.

Figure 6 presents the derivative thermogravimetric (DTG) curves of the raw materials (BW and PS) and the co-hydrochars produced at different PS blending ratios, illustrating their thermal decomposition behavior during combustion. Distinct degradation stages were observed among the samples, indicating differences in the thermal stability of the biomass, plastic, and the resulting co-hydrochars. For the raw BW, a small DTG peak below 150 °C corresponds to the evaporation of physically absorbed moisture [23,38]. The principal degradation stage occurred between approximately 200 and 350 °C, which is mainly attributed to the decomposition of hemicellulose and cellulose together with the gradual degradation of lignin and the release of volatile matter [23,28]. The relatively broad DTG peak reflects the complex composition of lignocellulosic biomass and the overlapping degradation of its major constituents. In contrast, raw PS exhibited a single, sharp DTG peak centered at approximately 400–420 °C, reflecting the thermal degradation of the polystyrene polymer backbone [31,32,33,39]. All co-hydrochars (10–50 wt% PS) also displayed a dominant DTG peak within the temperature range of approximately 400–450 °C. Compared with the raw BW, the maximum degradation temperature shifted toward higher temperatures, indicating that the Co-HTC process improved the thermal stability of the hydrochars.

Furthermore, the increasing PS proportion resulted in slightly broader degradation profiles while maintaining the principal degradation stage at higher temperatures. This behavior suggests that the carbonaceous matrix produced during Co-HTC became thermally more stable owing to the incorporation of aromatic carbon derived from PS. These results are consistent with the elemental analysis, FT-IR spectra, and Van Krevelen diagram, which demonstrated increased carbon content, reduced oxygen-containing functional groups, and progressive carbonization with increasing reaction temperature and PS content.

## 4. Conclusions

This study investigated the co-hydrothermal carbonization (Co-HTC) of brewery waste (BW) and polystyrene (PS) to produce carbon-rich hydrochar with improved fuel properties. The results demonstrated that both reaction temperature and PS content significantly influenced the physicochemical characteristics of the co-hydrochars. Increasing the reaction temperature from 220 to 260 °C and the PS proportion promoted carbon enrichment, reduced the H/C and O/C atomic ratios, and increased the higher heating value (HHV), indicating progressive carbonization of the hydrochar. The Van Krevelen analysis revealed that the co-hydrochars gradually shifted toward the lignite region with increasing reaction temperature, suggesting an enhanced degree of coalification. FT-IR analysis confirmed the progressive decomposition of oxygen-containing functional groups through dehydration and decarboxylation reactions, whereas SEM observations revealed the transformation from fractured fibrous biomass structures into compact carbonaceous aggregates. XRD analysis indicated that the co-hydrochars were predominantly composed of amorphous carbon with a low degree of structural ordering, while DTG analysis demonstrated improved thermal stability compared with the raw brewery waste. Overall, the results demonstrate that Co-HTC is an effective waste valorization strategy for simultaneously upgrading brewery waste and waste polystyrene into hydrochar with enhanced fuel characteristics. The combined effects of reaction temperature and PS incorporation provide an effective approach for improving hydrochar quality, highlighting the potential of Co-HTC for sustainable solid fuel production from mixed biomass and plastic wastes.

Although the present study focused on evaluating the fuel properties of the produced co-hydrochars, future work should investigate their combustion behavior, gaseous emissions, ash-related characteristics, and large-scale production to further assess their practical applicability as sustainable solid fuels.

## Figures and Tables

**Table 1:** Properties of raw feedstock and co-hydrochar.

Sample	Ultimate Analysis (wt%, Dry Basis)				Proximate Analysis (wt% Dry Basis)			Fuel Ratio
	C	N	H	O *	VM	FC	Ash	
BW	43.55 ± 0.80	6.15 ± 0.42	7.50 ± 0.32	42.80 ± 0.08	61.48	29.59	8.93	0.48
HC-BW 200	54.69 ± 0.87	7.22 ± 0.11	6.87 ± 0.10	31.22 ± 0.80	39.03	34.17	26.80	0.88
HC-BW 220	58.25 ± 0.64	6.63 ± 0.15	6.68 ± 0.21	28.44 ± 0.43	50.65	35.70	13.65	0.70
HC-BW 240	60.34 ± 1.57	6.86 ± 0.64	6.44 ± 0.40	26.36 ± 0.54	38.82	44.42	16.76	1.14
HC-BW 260	62.39 ± 0.57	6.64 ± 0.16	6.33 ± 0.23	24.64 ± 0.22	30.67	3.50	65.75	0.12
Co-HC 200	67.89 ± 0.42	6.87 ± 0.05	7.89 ± 0.04	17.35 ± 0.37	13.31	10.66	76.03	0.80
Co-HC 220	75.38 ± 0.43	3.32 ± 0.07	7.63 ± 0.10	13.67 ± 0.42	9.82	10.71	79.47	1.09
Co-HC 240	80.51 ± 0.32	2.01 ± 0.38	7.82 ± 0.17	9.66 ± 0.54	9.81	13.63	76.56	1.38
Co-HC 260	85.65 ± 0.17	1.88 ± 0.06	7.93 ± 0.10	4.54 ± 0.23	9.89	15.85	74.26	1.60

Note: * calculated by difference (mean ± SD; *n* = 3); VM—volatile matter, FC—fixed carbon.

## Data Availability

The data presented in this study are available on request from the corresponding author. The data are not publicly available due to institutional restrictions.
